# FISH molecular testing in cytological preparations from solid tumors

**DOI:** 10.1186/s13039-014-0056-9

**Published:** 2014-08-22

**Authors:** Paola Caria, Roberta Vanni

**Affiliations:** 1 Department of Biomedical Sciences, University of Cagliari – Cittadella Universitaria, 09042 Monserrato (CA), Cagliari, Italy

**Keywords:** FISH, Gene fusions, Cytology, Solid tumor, Gene promiscuity

## Abstract

Many of the exciting new developments in solid tumor molecular cytogenetics impact classical and molecular pathology. Fluorescence in situ hybridization to identify specific DNA target sequences in nuclei of non-dividing cells in solid neoplasms has contributed to the integration of molecular cytogenetics into cytology in spite of the remarkable promiscuity of cancer genes. Indeed, although it is a low-throughput assay, fluorescence in situ hybridization enables the direct disclosure and localization of genetic markers in single nuclei. Gene fusions are among the most prominent genetic alterations in cancer, providing markers that may be determinant in needle biopsies that are negative or suspicious for malignancy, and may contribute to the correct classification of the tumors. In view of the expanding use of fluorescence in situ hybridization in cytology, future challenges include automated sample evaluation and the specification of common criteria for interpreting and reporting results.

## Background

Various types of genetic alterations, as well as epigenetic phenomena, have been identified and are now considered important in the classification, prognosis, and treatment of cancer. Correlations between genomic instability and carcinogenesis have been extensively investigated, leading to the recognition of an increasing number of genetic abnormalities as a tumor driving force. Currently, several molecular approaches are available to investigate tumor cell pathobiology at different levels (chromosome, gene, gene expression). The predominant approaches include immunohistochemistry, fluorescence *in situ* hybridization (FISH), polymerase chain reaction, array-based and omics-based techniques [1]–[5]. The integration of results obtained using these platforms has been invaluable in clarifying genetic alterations associated with cancer and in interpreting the key role of the impaired signaling pathways. Gene gains and losses and gene disruptions by chromosome translocation, inversion, or deletion have been recognized as playing a pathogenetic role in many cancers. These exciting new developments in solid tumor molecular cytogenetics impact classical and molecular pathology, and an increasing number of chromosome markers have been integrated into World Health Organization tumor classifications [6]. Some of these markers are also relevant to selection of therapies targeting the protein products of gene fusions. In this scenario the impact of testing gene alterations by interphase FISH in material from needle biopsies and organic fluids has rapidly increased.

### Promiscuity: a false dilemma?

**Figure 1:**
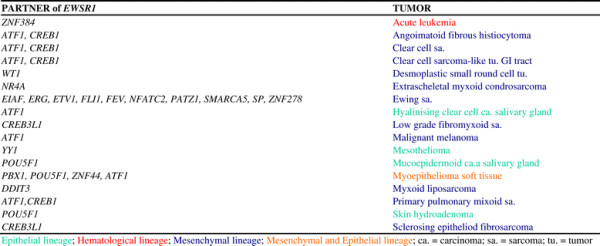
**Promiscuity of the** ***EWSR1*** **gene in malignancy.**

**Figure 2:**
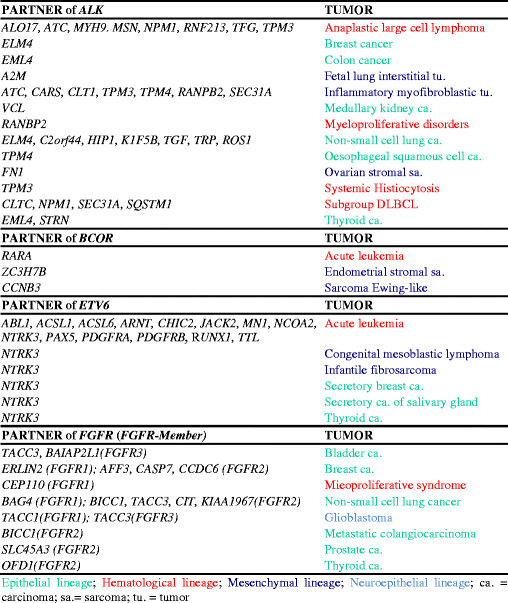
**Promiscuity of the** ***ALK,*** ***BCOR,*** ***ETV6,*** ***FGFR*** **genes in malignancy.**

**Figure 3:**
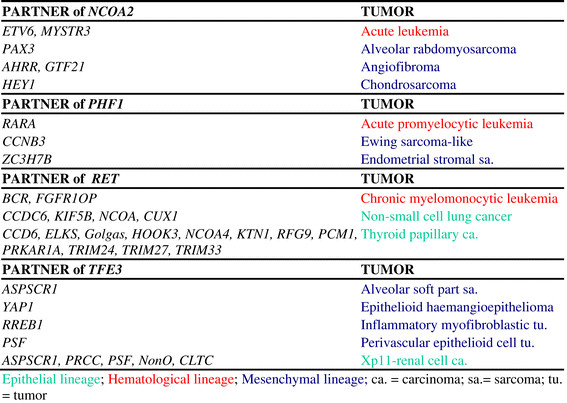
**Promiscuity of the** ***NCOA2,*** ***PHF1,*** ***RET,*** ***TFE3*** **genes in malignancy.**

The identification of a specific translocation in solid tumors dates back to 1983 when the t(11;22)(q24;q12) in Ewing’s sarcoma was first described [7]. It took nine years before the underlying gene fusion, *EWS/FLI1*[8] (today named *EWSR1/FLI1*), was discovered. The *EWS/FLI1* fusion was found to be closely associated with this type of sarcoma, and was thought to play a causal role in initiating the neoplastic process. Subsequent observations of variant translocations and the resulting *EWSR1* fusions with different partner genes in the same tumor entity disclosed the tip of an iceberg, paving the way for discovering the phenomenon of gene promiscuity in cancer. Indeed, the molecular cytogenetics of Ewing’s sarcoma family tumors (so called ESFT) and subsequently of other histologically unrelated soft tissue tumors, and finally of tumors arising in tissues of distinct embryological origin, demonstrated the ubiquitous involvement of the *EWSR1* gene in a wide spectrum of cancers, from sarcoma to carcinoma and to hematological malignancy (Figure 1). It is now clear that most structural gene alterations mediated by chromosome rearrangements (examples in Figures 2 and 3) [9],[10], drive malignancy in a variety of tumors of different hystogenetic types. Nevertheless, this evidence does not invalidate the role of gene fusions or deregulated genes as diagnostic tools. Indeed, the accumulated data prompted expansion of the understanding of gene promiscuity. For example, it is now clear that the *EWSR1* gene fuses with several genes mainly encoding transcriptional regulator factor families, resulting in deregulation of specific molecular pathways. These include ETS, homeobox-genes, zinc finger, and leucine-zipper transcription factor families. Disruption of these pathways may influence the pathogenesis of specific tumor types through a variety of activation mechanisms [11]. In spite of promiscuity, searching for gene involved in chromosome alterations leading to illicit shuffling of coding or regulatory sequences in cancer is becoming an invaluable approach in cytological investigations as well.

### Cytology and cytogenetics

**Figure 4:**
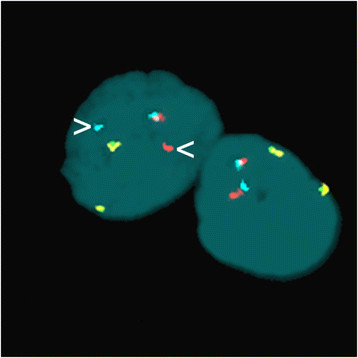
**Example of FISH in a cytological preparation.** A cytological preparation from thyroid fine needle aspiration was simultaneously hybridized with *RET* (labeled with Spectrum Aqua/Spectrum Red) and *PPARg* (labeled with Spectrum Green/Spectrum Gold respectively) split-apart probes. Broken *RET* is revealed by the split-apart centromeric aqua and telomeric red (arrowheads) probes. Contiguous dual-color signals indicate intact genes. Nuclei are counterstained with DAPI.

Based on the above scenario, the relationship between cytology and cytogenetics has become increasingly close, and the use of cytogenetics as an ancillary supplementary tool in cytological diagnosis has been introduced in the pathology sector. In particular, the leitmotif of the union of cytology and cytogenetics [12] is the need for a close collaboration between the two parts, since on one hand asking for a FISH test implies being aware of the rearrangement to be investigated (Figure 4), and on the other the cytogenetic result needs to be interpreted in the context of the cytological (and possibly clinical) observations. In addition, a FISH test is often used as a confirmatory tool since a negative result is not informative, both because unknown alterations cannot be excluded and the availability of tumor cells in cytological preparations may be limited.

Considering the introduction of systematic genomic testing for some tumors (such as lung and breast cancer) [13],[14], the consequent need for a correct evaluation of ratio value in the presence of genetic heterogeneity [15], and the growing demand for FISH tests in fine needle aspirations and organic fluids, two main challenges for the future can be foreseen: the implementation of automated FISH evaluation and the specification of common criteria for interpreting and reporting FISH results in as many tumor types as possible. A significant impediment to evaluating the ever increasing numbers of clinical FISH tests requested is imposed by the labor intensive nature of the assay, as each test requires scoring numerous interphase nuclei by double blind observation. Automated FISH, with strictly established parameters for standardization, could partly overcome these issues, although automation has yet to be perfected [16]. Specific recommendations and guidelines for FISH on tumors have been established within ACMG (American College of Medical Genetics and Genomics) [17] and E.C.A (European Cytogeneticists Association) [18]. On the other hand, common objective interpretation criteria for FISH on cytological preparations, as well as quality control and quality assurance policies, remain limited [13],[14], and require an extraordinary cooperative effort and interaction between cytogeneticists and cytologists. It would be desirable to convene expert advisory panels from scientific societies of clinical cytogeneticists and pathologists to establish evaluation criteria for the various tumors, based on expertise and a review of published literature, with a view to establishing common shared recommendations.

## Conclusions

Many of the exciting new developments of molecular cytogenetics are having a profound impact on classical and molecular cytology. The growing demand for cytological FISH tests highlights the need for the specification of common criteria for interpreting and reporting FISH results, for quality control and quality assurance policies, and for possible implementation of automated FISH evaluation.

## Competing interests

The authors declare that they have no competing interests.

## Authors’ contributions

PC and RV participated in commentary design and wrote the manuscript. They read and approved the final manuscript.

## References

[B1] Cai WW Mao JH Chow CW Damani S Balmain A Bradley A Genome-wide detection of chromosomal imbalances in tumors using BAC microarrays Nat Biotechnol 2002 20 393 396 10.1038/nbt0402-393 11923847

[B2] Maher CA Kumar-Sinha C Cao X Kalyana-Sundaram S Han B Jing X Sam L Barrette T Palanisamy N Chinnaiyan AM Transcriptome sequencing to detect gene fusions in cancer Nature 2009 458 97 101 10.1038/nature07638 19136943 PMC2725402

[B3] Hanash S Taguchi A The grand challenge to decipher the cancer proteome Nat Rev Cancer 2010 10 652 660 10.1038/nrc2918 20733593

[B4] Mainini V Pagni F Garancini M Giardini V De Sio G Cusi C Arosio C Roversi G Chinello C Caria P Vanni R Magni F An alternative approach in endocrine pathology research: MALDI-IMS in Papillary Thyroid Carcinoma Endocr Pathol 2013 24 250 253 10.1007/s12022-013-9273-8 24142502

[B5] Kanagal-Shamanna R Portier BP Singh RR Routbort MJ Aldape KD Handal BA Rahimi H Reddy NG Barkoh BA Mishra BM Paladugu AV Manekia JH Kalhor N Chowdhuri SR Staerkel GA Medeiros LJ Luthra R Patel KP Next-generation sequencing-based multi-gene mutation profiling of solid tumors using fine needle aspiration samples: promises and challenges for routine clinical diagnostics Mod Pathol 2014 27 314 327 10.1038/modpathol.2013.122 23907151

[B6] World Health Organization classification of tumours of soft tissue and bone 2013 IARC Press, Lyon

[B7] Turc-Carel C Philip I Berger MP Philip T Lenoir G Chromosomal translocation (11; 22) in cell lines of Ewing’s sarcoma C R Seances Acad Sci III 1983 296 1101 1103 6416622

[B8] Zucman J Delattre O Desmaze C Plougastel B Joubert I Melot T Peter M De Jong P Rouleau G Aurias A Thomas G Cloning and characterization of the Ewing’s sarcoma and peripheral neuroepithelioma t(11;22) translocation breakpoints Gene Chromosome Canc 1992 5 271 277 10.1002/gcc.2870050402 1283315

[B9] http://biome.ewha.ac.kr:8080/FusionGene/Search.jsp ᅟ: **ChimerDB 2.0-a knowledgebase for fusion genes updated**. In *ᅟ.* ; ᅟ [] 10.1093/nar/gkp982 PMC2808913 19906715

[B10] http://cancer.sanger.ac.uk/cancergenome/projects/cosmic/ᅟ: **COSMIC: Catalogue Of Somatic Mutations In Cancer**. In ***ᅟ***.; ᅟ. []

[B11] Cantile M Marra L Franco R Ascierto P Liguori G De Chiara A Botti G Molecular detection and targeting of EWSR1 fusion transcripts in soft tissue tumors Med Oncol 2013 30 412 10.1007/s12032-012-0412-8 23329308 PMC3586390

[B12] Dal Cin P Qian X Cibas ES The marriage of Cytology and Cytogenetics Cancer Cytopathol 2013 121 279 290 10.1002/cncy.21270 23288831

[B13] Lindeman NI Cagle PT Beasley MB Chitale DA Dacic S Giaccone G Jenkins RB Kwiatkowski DJ Saldivar JS Squire J Thunnissen E Ladanyi M Molecular testing guideline for selection of lung cancer patients for EGFR and ALK tyrosine kinase inhibitors. Guideline from the College of American Pathologists, International Association for the Study of Lung Cancer, and Association for Molecular Pathology Arch Pathol Lab Med 2013 137 828 860 10.5858/arpa.2012-0720-OA 23551194 PMC4162344

[B14] Wolff AC Hammond ME Hicks DG Dowsett M McShane LM Allison KH Allred DC Bartlett JM Bilous M Fitzgibbons P Hanna W Jenkins RB Mangu PB Paik S Perez EA Press MF Spears PA Vance GH Viale G Hayes DF Recommendations for human epidermal growth factor receptor 2 testing in breast cancer: American Society of Clinical Oncology/College of American Pathologists clinical practice guideline update Arch Pathol Lab Med 2014 138 241 256 10.5858/arpa.2013-0953-SA 24099077 PMC4086638

[B15] Bernasconi B Chiaravalli AM Finzi G Milani K Tibiletti MG Genetic heterogeneity in HER2 testing may influence therapy eligibility Breast Cancer Res Treat 2012 133 161 168 10.1007/s10549-011-1744-3 21901388

[B16] Pajor G Kajtár B Pajor L Alpár D State-of-the-art FISHing: automated analysis of cytogenetic aberrations in interphase nuclei Cytometry 2012 81 649 963 10.1002/cyto.a.22082 22696411

[B17] Mascarello JT Hirsch B Kearney HM Ketterling RP Olson SB Quigley DI Rao KW Tepperberg JH Tsuchiya KD Wiktor AE Working Group of the American College of Medical Genetics Laboratory Quality Assurance Committee. Section E9 of the American College of Medical Genetics technical standards and guidelines: fluorescence in situ hybridization Genet Med 2011 13 667 675 10.1097/GIM.0b013e3182227295 21738013

[B18] Hasting R Bown N Tibiletti MG Debiec-Rychter M Vanni R Espinet B van Roy N Roberts P van den Berg-de-Ruiter E Bernheim A Ylstra B Schoumans J Chatters S Zemanova Z Stevens-Kroef M Simons A Heim S Salido M Betts DR Guidelines for Cytogenetic Investigations in Tumours. E.C.A Newsletter 2014 34 7 18 http://e-c-a.eu http://e-c-a.eu 10.1038/ejhg.2015.35 PMC4795217 25804401

